# Supplementary figures and images for: Changes in instrumental activities daily living limitations and their associated factors according to gender in community-residing older adults: A longitudinal cohort study
Source: PLoS One. 2024 Jan 11;19(1):e0296796. doi: 10.1371/journal.pone.0296796 (PMC10783775; doi:10.1371/journal.pone.0296796)

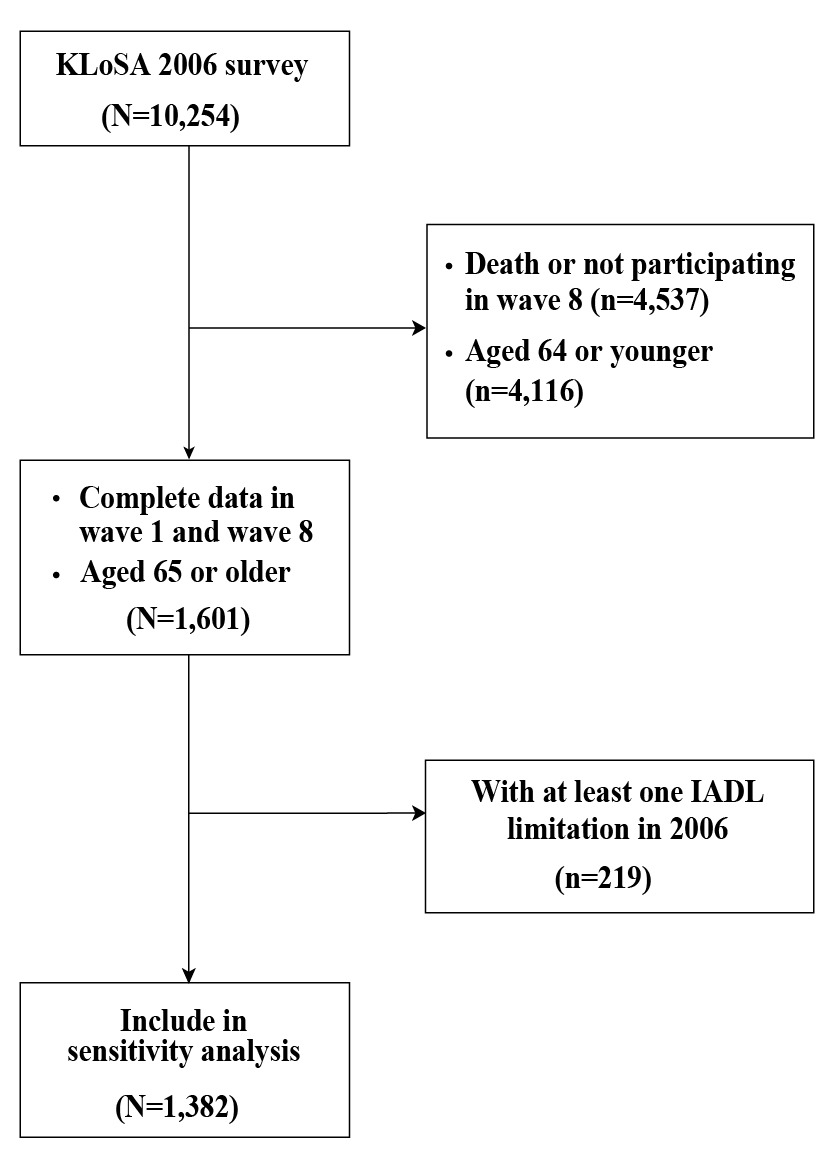

Supplement: S1 Fig — The sensitivity analysis was conducted to determine the influence of systemic selective attrition caused by follow-up loss during the data collection period on the factors of IADL limitations in older population. In the sensitivity analysis, 1,382 older adults were analyzed including people who incomplete questionnaires during wave 2 to 7. (TIF) [file pone.0296796.s001.tif]
