# Supplementary material for: Changes in instrumental activities daily living limitations and their associated factors according to gender in community-residing older adults: A longitudinal cohort study
Source: PLoS One. 2024 Jan 11;19(1):e0296796. doi: 10.1371/journal.pone.0296796 (PMC10783775; doi:10.1371/journal.pone.0296796)
Supplement: S1 Table — (DOCX) [file pone.0296796.s002.docx]

S1 Table. Univariate and multivariate logistic regression of total IADL limitations after 14 years including older adults not participated during wave 2-7 (N= 1,382)

| Variables^†^ | Total participants (N=1,382) | | Men (n=530) | | Women (n=852) | |
| --- | --- | --- | --- | --- | --- | --- |
|  | Unadjusted | Adjusted | Unadjusted | Adjusted | Unadjusted | Adjusted |
|  | OR (95% CI) | OR (95% CI) | OR (95% CI) | OR (95% CI) | OR (95% CI) | OR (95% CI) |
| Age (years) |  |  |  |  |  |  |
| 65-79 (ref) |  |  |  |  |  |  |
| 80-89 | 3.60(2.53-5.12)^**^ | 2.87(1.96-4.19)^**^ | 2.19(1.20-4.01)^**^ | 1.93(1.04-3.61) ^*^ | 4.07(3.00-7.37)^**^ | 3.25(1.98-5.33)^**^ |
| Living arrangement | |  |  |  |  |  |
| Living alone | 0.87(0.61-1.24) |  | 0.86(0.28-2.65) |  | 0.80(0.55-1.18) |  |
| Living with others (ref) | |  |  |  |  |  |
| Marital status |  |  |  |  |  |  |
| Married (ref) | |  |  |  |  |  |
| Not Married | 1.27(0.98-1.65) |  | 0.87(0.37-2.08) |  | 1.27(0.94-1.72) |  |
| Education (years) |  |  |  |  |  |  |
| 0-6 | 1.37(1.05-1.78)^*^ |  | 1.16(0.78-1.72) |  | 1.54(1.01-2.34)^*^ |  |
| More than 7 years (ref) | |  |  |  |  |  |
| Participate in social groups | |  |  |  |  |  |
| Yes (ref) |  |  |  |  |  |  |
| No | 1.09(0.85-1.40) |  | 1.10(0.71-1.70) |  | 1.06(0.78-1.45) |  |
| Regular exercise |  |  |  |  |  |  |
| Yes (ref) |  |  |  |  |  |  |
| No | 1.25(0.97-1.60) |  | 0.83(0.56-1.23) |  | 1.60(1.14-2.25)^**^ |  |
| Number of chronic diseases | |  |  |  |  |  |
| 1+ |  |  |  |  |  |  |
| 0 (ref) | 1.16(0.90-1.48) |  | 1.24(0.83-1.84) |  | 1.07(0.78-1.47) |  |
| Cognition |  |  |  |  |  |  |
| Dementia | 4.12(1.08-1.90)^**^ | 2.70(1.75-4.16)^**^ | 4.31(1.14-16.36)^*^ | 2.71(0.64-11.43) | 4.07(2.63-6.28)^**^ | 2.35(1.44-3.83)^**^ |
| MCI | 1.43(1.08-1.90)^**^ | 1.13(0.97-1.78) | 2.12(1.22-3.70) ^**^ | 2.03(1.14-3.59) ^*^ | 1.28(0.90-1.80) | 1.09(0.75-1.60) |
| Norma l (ref) |  |  |  |  |  |  |
| Depression |  |  |  |  |  |  |
| Yes | 1.23(0.87-1.74) |  | 1.57(0.80-3.08) |  | 1.09(0.72-1.64) |  |
| No (ref) |  |  |  |  |  |  |
| Trouble with pain | |  |  |  |  |  |
| Yes | 1.56(1.23-2.00)^**^ |  | 1.80(1.10-2.94)^*^ |  | 1.46(1.09-1.97)^*^ |  |
| No (ref) |  |  |  |  |  |  |
| Perceived health |  |  |  |  |  |  |
| Good (ref) |  |  |  |  |  |  |
| Bad | 1.63(1.22-2.19)^**^ |  | 1.57(1.02-2.41)^*^ |  | 1.63(1.08-2.46)^**^ |  |
| Perceived QoL | 0.99(0.99-1.00)^**^ |  | 1.00(0.99-1.01) |  | 0.99(0.98-0.99)^**^ |  |
| Falls (within 2years) | |  |  |  |  |  |
| Yes | 1.32(0.81-2.16) |  | 1.70(0.56-1.17) |  | 1.20(0.69-2.08) |  |
| No (ref) |  |  |  |  |  |  |
| FOF |  |  |  |  |  |  |
| Yes | 1.95(1.50-2.52)^**^ | 1.58(1.20-2.09)^**^ | 1.85(1.24-2.75)^**^ | 1.72(1.13-2.60)^*^ | 2.10(1.43-3.07)^**^ | 1.55(1.04-2.33)^*^ |
| No (ref) |  |  |  |  |  |  |
| Grip strength | 0.96(0.95-0.98)^**^ |  | 0.95(0.91-0.98)^**^ |  | 0.91(0.88-0.95)^**^ | 0.95(0.91-0.99)^**^ |
| BMI |  |  |  |  |  |  |
| Non-obesity (ref) |  |  |  |  |  |  |
| Obesity | 1.29(0.97-1.71) |  | 1.06(0.64-1.78) |  | 1.38(0.97-1.95) |  |

OR: odds ratio; CI: confidence interval; IADL: instrumental activities of daily living; MCI: mild cognitive impairment; QoL: quality of life; FOF: fear of falling; BMI: body mass index; **p*<0.05, ***p*<0.01, ^†^ Data of baseline (2006)
