# Supplementary material for: Changes in instrumental activities daily living limitations and their associated factors according to gender in community-residing older adults: A longitudinal cohort study
Source: PLoS One. 2024 Jan 11;19(1):e0296796. doi: 10.1371/journal.pone.0296796 (PMC10783775; doi:10.1371/journal.pone.0296796)
Supplement: Attachment — Submitted filename: Response to Reviewers.docx [file pone.0296796.s003.docx]

| Reviewers’ original comment | Author Response/ Page numbers and line numbers of changes made |
| --- | --- |
| Thank you for your comments! We have revised the manuscript based on the reviewer’s comments, please see the page number for each reviewers’ comment. We also got the professional editing service throughout the manuscript. | |
| Reviewer #1 Comment | Author Response |
| 1. The authors analyze only respondents who appeared in waves from 2004-2018. Was there systematic selective attrition? If I am understanding this correctly, about 23% of respondents were excluded? Was any sensitivity analyses conducted on who this group was? Could imputation be used for respondents with missing data? I think the mortality selection could be leading to the decrease finding for women in the first few waves. | Thank you for your comment! Unfortunately, we did not conduct sensitivity analysis in this study. We described this issue in limitation about careful interpretation of the results of this study (on page 22).  🡪Finally, systematic selective attrition caused by death and loss follow-up during the data collection period was not considered. In other words, there may be possibility of attrition bias in this study that does not reflect the characteristics of vulnerable group with the greatest limitation of IADL, so it is necessary to be careful in interpreting the results. In further study, it is suggested to conduct sensitivity analysis to minimize the attrition bias of the influencing factors of IADL limitation occurrence in this population. |
| 2. In the Health and Retirement Study, IADLs also have an option for “Do not do,” is that available here to analyze as well? | Thank you for your great comment! The IADL in KLoSA survey does not have an option ‘do not do’ response. We added the issue regarding on the response format in discussion (on page 18).  🡪The KLoSA survey does not include a response option of “do not perform” for any IADL item for any survey respondent in Korea. Sheehan et al. [20] argue that a “do not perform” response to IADL items allows many different responses by gender and cohort, potentially causing systematic biases in IADL limitations and potentially influencing changes in IADL limitations according to gender [12, 20]. Further research must identify the factors influencing IADL limitations according to gender in Korean older adults using an IADL index that includes a response option of “do not perform” for participants with no experience with certain IADLs. |
| 3. Should the tables be labeled as logistic models if they are predicting the sum? Sorry if I am not understanding this correctly. | Sorry for the confusion! We tried to added the longitudinal line in tables 2 and 3 to improve the readers’ better understanding the study result. |
| 4. Could table 2 be a figure instead? Or the figures included at the bottom replace it? | Yes, we agreed on your suggestion, and changed Table 2 to figure. We revised the section in main text (on page 10). Please see Figure 2.  🡪Total IADL scores increased over time, especially from wave 5, and changes were statistically significant in all groups [total participants: F = 104.82 (*p* < .001); men: F = 31.98 (*p* < .001); women: F = 74.36 (*p* < .001)].  🡪 Please see Figure 2. |
| 5. The figures are difficult to see clearly, could they be made larger? | Yes, we improved the resolution and size of the figures. Please see Figures 3 and 4. |
| 6. I could not tell if the data are available, could that be made more clear? | Sorry for the confusion. Since we used public data for this study, we changed data availability to ‘all data are fully available without restriction’. |
| Reviewer #2 Comment | Author Response |
| The manuscript addresses an important topic from a perspective that there are few studies exploring the changes in incidence of IADL limitations over a long period as well as identifying the related factors of IADL limitations according to gender. The authors use data from a broad and recognized population-based survey with a well know method. However, there are still some gaps that need to be addressed in order for the text to be suitable for publication. | Thank you for your comments! |
| 1. Data Availability: please detail which restrictions would apply | Sorry for the confusion. Since we used public data for this study, we changed data availability to ‘all data are fully available without restriction’. |
| 2. ABSTRACT  Conclusion: please insert a statement regard the changes in incidence of IADL limitations over the years. | Thank you for your comment! We added the statement in conclusion of the abstract.  🡪The incidence of IADL limitations gradually increased in all participants over a 14-year period. |
| 3. INTRODUCTION - It would be advisable to include a discussion of changes in the incidence/prevalence of IADL limitations over time according to the literature. | Thank you for your comment! We added the information regarding changes in the incidence/prevalence of IADL limitations over time using two literatures (on page 4).  🡪Limitations in IADL in older adults increase with age, with previous research reporting gradual increase over a 10-year period [4], as well as in a study with just three years of follow-up [8]. Most previous research examines changes in the incidence of IADL limitations in all segments of older populations together. Because incidence and limitations in IADL vary based on gender for different activities, it is important to assess these changes by gender to understand functional limitations among older adults. |
| 4. MATERIALS AND METHODS - Independent variables: 1) Please justify why to classify “regular exercise” as a sociodemographic variable.  2) Which is the category that includes the answer “1” for the “number of chronic diseases” variable? The description provided in that section does not correlates to Table 1 categories.  3) Please justify the reason for using the 2-year cutoff point in relation to the variable "experience of fall". | Thank you for your comment!   1) We classified “regular exercise” as health-related factors, and updated the method section (on page 8) and Tables 1, 2.   2) We calculated the number of chronic diseases (included hypertension, diabetes mellitus, cancer, pulmonary disease, liver disease, heart disease, cerebrovascular disease, psychiatric disease, and arthritis) by checking the chronic disease diagnosed by the doctor, and updated the method section (on page 8).  3) KLoSA survey were conducted every two years, and “fall experience” were asked for the experience during the past 2 years. We added the statement in methods section (on page 9). 🡪 Experience with falling was determined with the question, “Have you had a fall in the last two years?”, with answers of either “yes” or “no,” reflecting administration of the KLoSA every two years. |
| 5. RESULTS - I suggest to standardize the period in which the changes in IADL is assessed. In the abstract, the authors referred to “14 years” whereas in the Results, it appears a period of 12 years of changes. It seems there is an ambiguous interpretation of the period including or not including baseline wave (2006). | Thank your comment! We revised the observation period to 14 years based on baseline (2006, wave 1) throughout the main text. |
| - Please, cite the Table source of the data in the last paragraph of the Results section. | Thank your comment! We added table source (Table 3) (on page on 11). |
| - Table 2: please, insert subtitles for this Table. It would be advisable to include a column with the baseline records (2006) for the IADL total scores. | Thank your comment! We changed Table 2 to Figure 2 including the IADL total score of baseline (2006) in Figure 2-4. |
| - Tables 3 and 4: For a better visualization, I suggest a division between the columns containing data of the Total of the sample; Men; and Women. Furthermore, it is necessary to inform the year/wave of the data in Table titles. | Thank your comment! We revised Table 2 and 3 (page on 14-16). |
