# Supplementary material for: Changes in instrumental activities daily living limitations and their associated factors according to gender in community-residing older adults: A longitudinal cohort study
Source: PLoS One. 2024 Jan 11;19(1):e0296796. doi: 10.1371/journal.pone.0296796 (PMC10783775; doi:10.1371/journal.pone.0296796)
Supplement: Attachment — Submitted filename: Response to Reviewers_final.docx [file pone.0296796.s004.docx]

| Editor’s comment | Author Response/ Page numbers and line numbers of changes made |
| --- | --- |
| Thank you for your comments! We have revised the manuscript based on the editor’s comments. | |
| Editor Comment | Author Response |
| I have re-read the manuscript submitted as Revision 2.  The previous reviewers had already advised that we accept the manuscript, but each made a small suggestion, which I included in the previous editor's decision, so that the acceptance was conditional on addressing them. | Thank you very much for your comment to improve the quality of the manuscript and we have added some thoughtful contents. |
| The first suggestion was that the authors perform a sensitivity analysis to better understand the attrition from the sample.  Although the authors have added a table to the supplemental files, the results are not described in the text, and the table title is unclear.  The N for the table is 1382.   The N for the analysis is 1230. | Thank you very much for your valuable comment.  We added the following information (on page 10) regarding the purpose of sensitivity analysis and along with a supplementary figure (Figure S1) detailing the participants included.  Additionally, we have added the specific results of the sensitivity analysis and the strength of the sensitivity analysis on page 12 and 23. |
| The Supplemental table's title suggests that it is an analysis of data from those missing from the final sample because of missing data in waves 2-7, but the language is unclear. Moreover, the added text describing the results of the sensitivity analysis do not refer to attrition at all.  Please explain the data sources and purpose of the analysis. | Thank you for your comment.  We changed the title of Supplemental table to ‘Univariate and multivariate logistic regression of total IADL limitations after 14 years including older adults not participated during wave 2-7 (N= 1,382)’. |
| The second suggestion was to change the language for the covariate of obesity.  It seems that no change was made, only a reference to the World Health Organization's categorization was made.  Please explain your decision. | We apologize for any confusion caused by the description the covariate of obesity. Actually, we did not change the language for the covariate of obesity. To make clear, we added the classification of WHO Asian Criteria of Obesity in detail on page 9. Thank you! |
